# Supplementary material for: Single-cell lineage tracing identifies hemogenic endothelial cells in the adult mouse bone marrow
Source: eLife. 2026 Apr 27;15:RP109553. doi: 10.7554/eLife.109553 (PMC13120821; doi:10.7554/eLife.109553)
Supplement: Supplementary file 1. [file elife-109553-supp1.docx]

**Supplementary file 1. Distribution of Polylox barcodes among bone marrow cell populations.**

| cell_type | Total cells | Cells with barcodes (pGen < 1) | Cells with true barcodes  (pGen < 1e-6) | Cells with not true barcodes  (pGen ≤ 1e-6 < 1) |
| --- | --- | --- | --- | --- |
| B cells | 5977 | 344 | 73 | 271 |
| BM resident macrophages | 547 | 18 | 5 | 13 |
| Dendritic Cells | 6471 | 235 | 26 | 209 |
| Doublets | 2607 | 87 | 17 | 70 |
| Endothelial cells | 21168 | 828 | 169 | 659 |
| Erythroblasts and erythroid precursors | 1809 | 78 | 13 | 65 |
| GMP | 3980 | 121 | 11 | 110 |
| Granulocytes | 37931 | 1674 | 317 | 1357 |
| HSPC | 1257 | 71 | 14 | 57 |
| Mesenchymal type | 2544 | 115 | 31 | 84 |
| Monocytes | 4840 | 281 | 40 | 241 |
| Plasma cells | 824 | 49 | 8 | 41 |
| T cells | 1580 | 52 | 3 | 49 |
| pDCs | 2018 | 119 | 11 | 108 |

Number of bone marrow cells annotated based on marker gene expression containing any Polylox barcode, a true Polylox barcode (defined as pGen < 1e-6), and a not true Polylox barcode (defined as pGen ≤ 1e-6 < 1).
